# Diabetes and Diabetes Care among Nonobese Japanese-Americans: Findings from a Population-Based Study

**DOI:** 10.1155/2019/3650649

**Published:** 2019-06-02

**Authors:** Karen Kobayashi, Keith Tsz-Kit Chan, Adity Roy, Mushira Mohsin Khan, Esme Fuller-Thomson

**Affiliations:** ^1^Department of Sociology and Institute on Aging and Lifelong Health, University of Victoria, Victoria, BC V8W 3P5, Canada; ^2^School of Social Welfare, University at Albany SUNY, Richardson, Rm 277, USA; ^3^Institute for Life Course & Aging, University of Toronto, Toronto, ON, Canada; ^4^Institute for Life Course & Aging, Factor-Inwentash Faculty of Social Work, Cross-Appointed to the Faculty of Medicine University of Toronto, 246 Bloor St. W., Toronto, ON, Canada

## Abstract

**Objectives:**

The objectives of this study are as follows: (1) to determine the prevalence of diabetes among nonobese Japanese-Americans and to determine the adjusted odds of diabetes among nonobese Japanese-Americans compared to non-Hispanic Whites (NHWs); (2) to identify the risk factors associated with having diabetes in a large sample of nonobese Japanese-Americans; and (3) to determine the prevalence and adjusted odds of diabetes management behaviors among nonobese Japanese-Americans with diabetes in comparison to NHWs with diabetes.

**Methods:**

The combined 2007-2016 waves of the adult California Health Interview Survey (CHIS) were used to analyze a nonobese (BMI<30) sample of 2,295 Japanese-Americans and 119,651 NHWs. Chi-square and logistic regression analyses were performed using Stata.

**Results:**

The findings of this representative community study of nonobese Californians indicate that the prevalence of diabetes among Japanese-American respondents was higher than their NHW counterparts (8.0% versus 4.5%). Prevalence increased markedly with age; one-quarter of nonobese Japanese Americans aged 80 and older had diabetes.

**Conclusions:**

The prevalence of diabetes among nonobese Japanese-Americans is significantly higher than that among NHWs. There is an urgent need to develop appropriate intervention and prevention approaches with lifestyle modification specifically targeted towards nonobese Japanese-Americans.

## 1. Introduction

Although Asian-Americans are the fastest growing ethnic/racial minority groups in the United States with a growth rate that is more than four times that of the total population, this group remains underrepresented in most epidemiological studies [1–3]. Moreover, studies including Asian-Americans typically aggregate multiple subethnic groups under one large category [4]. According to the U.S. Census Bureau, the Asian-American category includes individuals with origins from Eastern Asia (Korea, China, Japan, and Mongolia), Southeast Asia (Malaysia, Thailand, Cambodia, the Philippine Islands, Vietnam, Singapore, Indonesia, Laos, etc.), and/or the Indian subcontinent (Pakistan, India, Bangladesh, Nepal, Bhutan, and Sri Lanka) [2]. These subethnic groups are diverse in nature, each with a unique language, history, and culture [2]. Furthermore, there are significant differences in physiology and body composition among the Asian-American subethnic groups [2]. Aggregating such a large and diverse population under one category of Asian-Americans masks the heterogeneity in lifestyle and health practices, and in the prevalence of chronic conditions. These differences need to be taken into consideration when developing prevention and intervention approaches to target specific subethnic groups, especially for chronic disease management.

The population of Asian residents in the United States in 2016 was approximately 21.4 million, including 1.5 million Japanese-Americans [5]. Research suggests that there are significant differences in health outcomes and disease prevalence between Japanese-Americans and their white counterparts as well as other Asian-American groups. Diabetes is a chronic condition that has been shown to be more prevalent in Japanese-Americans than in the general U.S. population or in Japanese citizens [6]. A large cross-sectional study exploring the gender and ethnic differences in the prevalence of type 2 diabetes among Asian subgroups in California concluded that Japanese-Americans had one of the highest age-adjusted diabetes prevalence (11.8%) among men, as compared to their peers from the following groups: non-Hispanic White (NHW), South Asian, Chinese, Korean, Vietnamese, and Mexican [7].

The objectives of this study are as follows: (1) to determine the prevalence of diabetes among nonobese Japanese-Americans and to determine the adjusted odds of diabetes among nonobese Japanese-Americans compared to NHWs; (2) to identify the risk factors associated with having diabetes in a large sample of nonobese Japanese-Americans; and (3) to determine the prevalence and adjusted odds of diabetes management behaviors among nonobese Japanese-Americans with diabetes in comparison to NHWs with diabetes.

## 2. Methods

### 2.1. Data Source

As has been described elsewhere [8, 9], data were taken from the combined 2007, 2009, 2011, 2013, 2014, 2015, and 2016 waves of the adult California Health Interview Survey (CHIS). Using a multistage sampling design, CHIS collected survey data using random-digit-dialing (RDD) with landline and cellular samples for respondents for a representative sample of Californian adults. Interviews were conducted in five languages: English, Spanish, Chinese (Mandarin and Cantonese dialects), Vietnamese, and Japanese.

### 2.2. Sample

The total combined sample of adult respondents over 18 years old from 2007, 2009, 2011, 2013, 2014, 2015, and 2016 waves of the CHIS data included 2,634 Japanese-Americans and 156,388 NHWs. Approximately 10.0% of Japanese-Americans and 7.1% of NHWs from the full sample had diabetes. Almost seven in 10 Japanese-Americans (68.3%) with diabetes are in the nonobese BMI range (i.e., Body Mass Index (BMI) <30). This contrasted with 49.6% of NHWs with diabetes and the same BMI range.

For this paper, the final study sample included 121,946 respondents with BMI less than or equal to 29.99 (hereafter referred to as “nonobese BMI”), of whom 2,295 were Japanese-Americans and 119,651 were NHWs.

### 2.3. Measures

#### 2.3.1. Identification of Individuals with BMI <30

Individuals were asked in the CHIS survey, “How tall are you without shoes?” to determine height, and “How much do you weigh without shoes?” to determine weight. BMI was then calculated by dividing weight in kilograms by the square of height in meters.

#### 2.3.2. Identification of Individuals with Diabetes

The question regarding diabetes was found in the CHIS survey, “Other than during pregnancy, has a doctor ever told you that you have diabetes or sugar diabetes?” Diabetes or sugar diabetes in this case refers to either type 1 or type 2 diabetes mellitus.

#### 2.3.3. Demographic Characteristics

The following demographic variables were examined: (1) Japanese* vs.* NHW ethnicity; (2) sex; (3) age (18 and over); (4) attainment of postsecondary degree; (5) poverty (0 to 99% of Federal Poverty Level versus 100% or more)—this variable takes into account household income in the context of household composition; and (6) immigrant status (foreign-born or not).

#### 2.3.4. Identification of Health-Related Variables

For cigarette smoking, respondents were asked “Altogether, have you smoked at least 100 or more cigarettes in your entire lifetime?” (coded never* vs.* ever).

Due to changes in questions among the waves, we created a new variable “moderate physical activity” which was defined as follows: From the 2007 dataset, the variable was constructed from the question “During the last 7 days, did you do any moderate physical activities in your free time for at least 10 minutes?” From the 2009 and 2016 datasets, we used the question “Sometimes you may walk for fun, relaxation, exercise, or to walk the dog. During the past 7 days, did you walk for at least 10 minutes for any of these reasons?” (coded yes* vs.* no).

BMI was coded into two categories to control the differences in body weight profiles, as BMI less than 25 and BMI of 25 to 29.99.

#### 2.3.5. Diabetes Health Management Variables

The following diabetes health management variables were examined: (1) regular eye exams; (2) regular foot exams; (3) regular hemoglobin A1C checks; and (4) confidence in managing diabetes. For regular eye exams, respondents were asked, “When was the last time you had an eye exam in which the pupils were dilated?” Responses of “within the past month,” “1 to 12 months ago,” and “1 to 2 years ago” were coded as optimal eye care, and “more than 2 years” coded as suboptimal eye care. For regular foot exams, respondents were asked, “About how many times in the last 12 months has a doctor checked your feet for any sores or irritations?” Responses of 1 to 52 were coded as optimal foot exam, and 0 coded as suboptimal. For regular A1C glycated hemoglobin checks, respondents were asked, “About how many times in the last 12 months has a doctor or other health professional checked you for hemoglobin “A one C?” Responses of 2 or more times a year were coded as optimal, and less than 2 times as suboptimal. For confidence in managing diabetes, respondents were asked, “How confident are you that you can control and manage your diabetes?” Responses of “very confident” and “somewhat confident” were coded as confident, and “not too confident” and “not at all confident” were coded as not confident. For insulin dependence, respondents were asked, “Are you now taking insulin?” Responses of “yes” were coded as insulin dependent, and “no” coded as not insulin dependent.

### 2.4. Data Analyses

Chi-square analyses were used to compare nonobese Japanese-Americans and NHWs with respect to the prevalence of diabetes (Objective 1a) and a range of sociodemographic characteristics and health behaviors. A multivariate logistic regression analysis of diabetes status was conducted to determine the odds of diabetes for nonobese Japanese-Americans in comparison to NHWs from California (Objective 1b).

In the Japanese subsample, chi-square tests compared those with and without diabetes (Objective 2a). A logistic regression analysis of diabetes status was conducted among nonobese Japanese-Americans, which included sociodemographic characteristics and health behaviors (Objective 2b).

Further chi-square tests and logistic regression analyses were conducted for nonobese Japanese-Americans and NHWs who have diabetes, with respect to health management behaviours, such as regular eye exams, foot exams, haemoglobin A1C levels, and confidence in managing diabetes (Objective 3). This set of analyses controlled for insulin use, sociodemographic characteristics and overweight status.

Survey design weights using jackknife replication techniques were employed in the calculation of percentages, standard errors, and odds ratios (OR) to adjust for the probability of selection. A final household weight and 560 replicate weights were created from the 2007, 2009, 2011, 2012, 2013, 2014, 2015, and 2016 data, using procedures outlined on the CHIS website [6]. All analyses within this article were performed using Stata 15 (StataCorp, College Station, Texas, USA). All sample sizes are provided in their unweighted form.

## 3. Results

### 3.1. Descriptive Variables Characteristic

As shown in Table 1, among nonobese Californians aged 18, the prevalence of diabetes among Japanese-American respondents was higher than their NHW counterparts (8.0% vs. 4.5%). This was despite the fact that Japanese-Americans in the sample were similar in age to NHWs and had fewer risk factors for diabetes. Japanese-Americans were also more likely to have a postsecondary degree, were less likely to live in poverty, were less likely to smoke, and had lower BMI compared to their NHW peers. However, Japanese-Americans were less likely to exercise than NHW, which is a risk factor for diabetes. With respect to other characteristics, it is important to note that three-quarters of Japanese-Americans were born in the US (74.5%) and they had a higher prevalence of women in the sample than NHWs (58.7%* vs.* 51.8%).

Once a range of sociodemographic characteristics and health behaviors were taken into account, the adjusted odds ratio (OR) of diabetes was double for nonobese Japanese-Americans compared to NHWs (OR = 2.08, 95% CI: 1.49, 2.90) (see Table 1). In the general California population, men had 50% higher odds of diabetes compared to women (OR = 1.50, 95% CI: 1.32, 1.70). Each decade older in age was associated with 61% higher odds of diabetes (OR = 1.61, 95% CI: 1.55, 1.68). Those without a college degree had 41% higher odds of diabetes than those with a degree (OR = 1.41, 95% CI: 1.24, 1.60). Respondents living in poverty had 51% higher odds of diabetes compared to those above the poverty line (OR = 1.51, 95% CI: 1.17, 1.93). Overweight Californians had 81% higher odds of diabetes compared to those with a BMI less than 25 (OR = 1.81, 95% CI: 1.57, 2.08). Ever smokers had 19% higher odds of diabetes (OR = 1.19, 95% CI: 1.02, 1.38) compared to never smokers. Those who did not engage in moderate exercise had 14% higher odds of diabetes (OR=1.14, 95% CI: 1.01, 1.30). Foreign-born status had no statistically significant association with the odds of diabetes for those with BMI<30.

### 3.2. Factors Associated with Diabetes among Nonobese Japanese-Americans

As indicated in the bivariate analyses restricted to nonobese Japanese-Americans only (see Table 2), those with diabetes were more than two decades older than those without diabetes. The prevalence of diabetes was much lower among those with a postsecondary degree compared to those without a degree (6.2%* vs.* 11.4%), among never smokers in comparison to ever smokers (5.4%* vs.* 14.1%), and among those with a normal BMI in comparison to those who were overweight (6.3%* vs.* 11.7%). In the logistic regression analysis controlling for a wide range of risk factors at the same time, age was the only variable which was associated with higher odds for diabetes. The fact that the odds of diabetes almost doubled for each decade of age in the Japanese nonobese sample (OR = 1.91, 95% CI: 1.52, 2.39) underlines the importance of age in the development of diabetes. We conducted additional bivariate analyses which demonstrated that the prevalence of diabetes among nonobese Japanese-Americans increased markedly with age from 1.2% in those under age 50 to 7.4% for those aged 50-64 to 19.6% for those aged 65 to 79 and 24.6% for those aged 80 and over.

### 3.3. Prevalence and Adjusted Odds of Diabetes Management Behaviors

As shown in Table 3, Japanese-Americans had a significantly higher prevalence than NHWs of having had an eye exam within the preceding two years, although both ethnicities were largely in compliance (95.2% vs. 87.1%; p=.002). After adjustment for age, sex, education, poverty, and BMI category and insulin dependence, NHWs had 65% lower odds of having had an eye exam in the optimal time period in comparison to Japanese-Americans (OR=0.35, 95% CI: 0.17, 0.74) (please see Table 4).

Almost all Japanese-Americans and NHW were confident in their ability to control and manage their diabetes (95.9%* vs.* 94.7%, p=0.71). Although Japanese-Americans and NHWs had a comparable prevalence of both having had a foot exam in the preceding year (69.6%* vs.* 73.4%, p=0.67) and having had their hemoglobin A1C levels checked at least twice in the preceding year (73.8%* vs.* 69.3%, p=0.58), these important routine preventive interventions/checks could have been improved, i.e., increased in frequency.

## 4. Discussion

The findings of this representative community study of nonobese Californians suggest that the prevalence of diabetes among Japanese-Americans is substantially higher than their NHW counterparts (8.0% vs. 4.5%). This finding is surprising given the fact that Japanese-Americans were more likely to have a postsecondary degree and less likely to smoke and had lower BMI compared to their NHW counterparts, and all of these factors are associated with a lower likelihood of diabetes. Even after taking these factors, in addition to age, gender, exercise level, and nativity, into account, Japanese-Americans had double the odds of diabetes compared to NHWs. Three-quarters of Japanese-Americans were born in the US, a higher percentage than in most Asian-American groups.

Our finding of a higher prevalence of diabetes among Japanese-Americans compared to NHWs is consistent with previous studies' findings on ethnic differences in diabetes prevalence across the whole BMI spectrum [6, 7]. Obesity is a major risk factor for the development of diabetes. The prevalence of obesity in Asian-Americans (10.8%) is significantly lower than that in all U.S. adults (34.9%) [10]. It is not surprising that the prevalence of diabetes in our study among the nonobese Japanese-Americans and NHWs (8.0%* vs.* 4.5%, respectively) was lower than that found in another population-based study that included participants across the whole BMI spectrum (men: 11.8%* vs.* 6.1%; women: 7.6%* vs.* 4.9%, respectively) [7].

The study's findings of such a high prevalence of diabetes in this nonobese sample are supportive of other findings indicating that Asian-Americans have an increased risk of diabetes despite having a mean BMI that is significantly lower than the typical cut-point of 25 that is indicative of overweight status [11, 12]. According to recent research, the risk of developing diabetes among Asians increased markedly at BMI values below the usual cut-point to identify those who are overweight (25 kg/m^2^) [11]. According to a position statement published by the American Diabetes Association, the recommended BMI cut-point for diabetes screening in the US for the Asian-American population was reduced to 23 kg/m^2^[2]. This recommendation was later incorporated into the “Standards of Medical Care in Diabetes” in 2015 [13].

The increased prevalence of diabetes in the current analysis among Japanese-Americans with BMI less than 30 kg/m^2^ can possibly be due to the difference in body fat distribution. According to a prospective study exploring diabetes incidence among Japanese-Americans, the development of type 2 diabetes in Japanese-Americans is preceded by greater visceral adiposity and is independent of family history of diabetes, sex, total and regional adiposity, and glycemia and correlates to insulin resistance and secretion [14]. A potential mechanism explaining the association between visceral fat and diabetes risk is the fact that visceral fat is more lipolytic which may lead to hepatic delivery of excessive fatty acid deposits, resulting in insulin resistance and hyperglycemia [14]. This process can be prevented by exercise and dietary intervention. However, our study indicated that more than one-third of Japanese-Americans were not exercising even at a moderate level. One study exploring the effect of lifestyle modification in Japanese-Americans with impaired glucose tolerance concluded that regular participation in endurance exercise and adherence to a diet that is low in saturated fat resulted in improved body fat distribution, body composition, and BMI in Japanese-Americans [15]. The results of this study suggest that improved outreach, education, and long-term lifestyle modifications may be effective in delaying or preventing type 2 diabetes in Japanese-Americans. However, even when we took exercise, BMI, and other risk factors into account, the adjusted odds ratio (OR) for diabetes was double for Japanese-Americans compared to NHWs suggesting that other, as yet unidentified, mechanisms may be at play.

This study concluded that in the general sample (see Table 1) older age, lack of a college degree, being male, living in poverty, being overweight (BMI from 25 to 29.99) as opposed to normal weight (BMI <25), and ever smoking were associated with higher odds for diabetes, as has been documented elsewhere [16–21]. We found that men had 50% higher odds for diabetes in this nonobese BMI group (OR = 1.50, 95% CI: 1.32, 1.70). This is consistent with previous studies exploring the gender differences in diabetes prevalence. Globally, men were reportedly affected with diabetes significantly more than women in 2013 [21].

We also found in the larger sample and in the Japanese-American subsample that each decade of age was associated with much higher odds of diabetes (OR = 1.91, 95% CI: 1.52, 2.39). Older age is a well-known contributor to diabetes in the general population [17, 18]. Additionally, in the general and Japanese-American samples, the prevalence of diabetes was higher among those without a college degree than among those with a postsecondary degree. A cross-sectional study exploring the association between educational attainment and diabetes prevalence among adults in US concluded that individuals with high school education were approximately twice as likely to report having diabetes compared to those with a bachelor's degree [20]. This suggests that educational attainment may promote a healthy lifestyle with adequate nutrition. The lack of a statistically significant link between educational attainment and diabetes among Japanese-Americans in the logistic regression analysis may be partially due to a lack of power, since Japanese-Americans were significantly more likely to have a college degree than NHWs and the relationship was significant in the bivariate analyses.

In contrast to the findings in the general sample, physical activity was not significantly associated with the odds of diabetes for nonobese Japanese Americans in our study. In the general sample, those who had ever smoked were more likely to have diabetes than never smokers. Cigarette smoking has been associated with low secretion of insulin along with high insulin resistance which may lead to diabetes [19]. In our subsample of Japanese Americans, a significant association between smoking and diabetes was found for the bivariate analyses, but not the logistic regression analysis. This may also be due to the limited statistical power in the study and the low rates of smoking in our Japanese subsample with only 30% ever smokers. A multiethnic cohort study including African-American, Japanese-American, Latino, Native Hawaiian, and white men and women reported that Japanese-Americans have the lowest percentage of smokers among women and significantly lower percentage of current smokers than African-Americans and Native Hawaiians among men [22].

Finally, we have found that living in poverty was associated with a higher prevalence of diabetes in the general population but not among Japanese-Americans. Low income individuals are more likely to develop diabetes [16]. The risk factors associated with higher incidence of diabetes among those living in poverty include unhealthy eating behaviors partially because of the caloric dense nature of inexpensive, highly processed, nutrient-poor food, obesity, hazardous home environment, and stress [16]. Moreover, the adverse effects of poverty on physical and mental health have been shown to be evident at young ages and persistent across the life course, and this has been documented across different cultures [23]. The lack of a statistically significant link between poverty status and diabetes among Japanese-Americans may be partially due to culturally based protective factors that were unaccounted for in this study.

Although there is evidence of Japanese-Americans having an incidence of type 2 diabetes that is approximately twice that of the Japanese living in Japan [24], our study results suggest that foreign-born status was not significantly associated with the odds of diabetes among nonobese Japanese Americans.

A positive finding of this study was that, once diagnosed, it appears that Japanese-Americans receive comparable levels of diabetes care as NHW and, in the case of regular eye exams, even appear to be at a slight advantage. Similarly, in a study comparing South Asian-Americans and NHWs using data from combined waves of the CHIS, despite the former group's higher propensity for diabetes, there were no significant differences between the two groups in the level of optimal care received by those with diabetes [9]. Although Japanese-Americans and NHWs were equally likely to receive foot exams and hemoglobin A1C checks, both groups would benefit from targeted outreach since more than a quarter of respondents had not received these treatments at an optimal frequency in the preceding year.

## 5. Conclusions

In summary, our findings suggest that the prevalence and adjusted odds for diabetes among nonobese Japanese-Americans are approximately double that of NHWs. To our knowledge, this study is the first large population-based study to examine the prevalence of diabetes among nonobese Japanese-Americans. Our results suggest that older Japanese-Americans are at high risk of developing diabetes. This highlights an urgent need for front-line healthcare professionals as well as health policy makers to develop appropriate intervention and prevention approaches with lifestyle modification specifically targeted towards nonobese Japanese-Americans as they get older. As previously discussed, preventative approaches with lifestyle modifications have been shown to be effective in delaying the onset of diabetes among high-risk Japanese-Americans. Additionally, lowering the BMI threshold for diabetes screening among Japanese-Americans is an important initiative. There is a need to improve primary prevention through lifestyle modifications for Japanese-Americans with prediabetes symptoms in order to prevent or delay the development of diabetes.

Finally, of relevance for preventive medicine, it is important for family physicians and other healthcare professionals to note that there is still substantial room for improvement for both Japanese-Americans and NHWs with regard to the frequency of provision of foot exams and hemoglobin A1C checks.

## Figures and Tables

**Table 1:** Descriptive variables characteristics and odds of having diabetes (OR) of nonobese Japanese-American and non-Hispanic White cohorts from the 2007, 2009, 2011, 2012, 2013, 2014, 2015, and 2016 CHIS (n=121,946).

	White (n = 119,651)	Japanese (n = 2,295)	p-value		Complete Model OR (95% CI)	p-value
Without Diabetes	95.5%	92.0%	0.0003	Non-Hispanic White (Ref.)	1.00	
With Diabetes	4.5%	8.0%		Japanese Americans	2.08 (1.49, 2.90)	<0.001
***Demographics***				***Demographics***		
**Gender**				**Gender**		
Male	48.3%	41.3%	0.02	Female (Ref.)	1.00	
Female	51.8%	58.7%		Male	1.50 (1.32, 1.70)	<0.001
**Age**				**Age**		
Mean (95% CI)	49.23 ± 0.23 (49.0-49.5)	49.9 ± 1.03 (47.9-51.9)	0.86	**(by Decade) †**	1.61 (1.55, 1.68)	<0.001
***Socioeconomic Status***				***Socioeconomic Status***		
**Education**				**Education**		
No Post-Secondary Degree	43.8%	34.0%	<0.0001	Post-Secondary Degree (Ref.)	1.00	
Post-Secondary Degree	56.2%	66.0%		No Post-Secondary Degree	1.41 (1.24, 1.60)	<0.001
**Poverty Level**				**Poverty Level**		
Not In Poverty	92.7%	93.1%	0.866	Not In Poverty (Ref.)	1.00	
In Poverty	7.3%	6.9%		In Poverty	1.51 (1.17, 1.93)	<0.001
***Health Variables***				***Health Variables***		
**Lifetime Smoking**				**Lifetime Smoking**		
Less than 100 Cigarettes	57.6%	69.7%	<0.0001	Less than 100 Cigarettes (Ref.)	1.00	
100 or more Cigarettes	42.4%	30.3%		100 or more Cigarettes	1.19 (1.02, 1.38)	0.02
**Nativity**				**Nativity**		
US-born	89.9%	74.5%	<0.0001	US-born (Ref.)	1.00	
Foreign-born	10.1%	25.5%		Foreign-born	1.03 (0.83, 1.28)	0.79
**Exercise**				**Exercise**		
Less than Moderate Exercise	28.7%	36.1%	0.003	Less than Moderate Exercise	1.14 (1.01, 1.30)	0.04
Moderate Exercise	71.3%	63.9%		Moderate Exercise (Ref.)	1.00	
**Body Mass Index (BMI)**				**Body Mass Index (BMI)**		
BMI< 25 (Ref)	55.1%	68.6%	<0.0001	BMI< 25 (Ref)	1.00	
BMI >=25, <30	44.9%	31.4%		BMI >=25, <30	1.81 (1.57, 2.08)	<0.001

**†** Age was divided by 10 and can be interpreted as odds for diabetes by decade.

All variables in the table were included in the logistic regression analysis.

**Table 2:** Descriptive characteristics and odds of having diabetes (OR) of nonobese Japanese-American adults with and without diabetes (n=2,295). Source: 2007, 2009, 2011, 2012, 2013, 2014, 2015, and 2016 CHIS.

	Without Diabetes (n = 2,026)	With Diabetes (n = 269)	p-value	Fully Adjusted Model OR (95% CI)	p-value
***Demographics***					
**Gender**					
Male	91.5%	8.5%	0.72	1.30 (0.57, 2.95)	0.54
Female (Ref.)	92.3%	7.7%		1.00	
**Mean Age** (95% CI)	48.1±1.07 (46.0-50.2)	70.4±2.50 (65.5, 75.3)	<0.0001	1.91 (1.52, 2.39)	<0.001
***Socioeconomic Status***					
**Education**					
No Post-Secondary Degree	88.6%	11.4%	0.04	0.93 (0.44, 1.94)	0.84
Post-Secondary Degree (Ref.)	93.8%	6.2%		1.00	
**Poverty Level**					
Not In Poverty (Ref.)	91.9%	8.1%	0.69	1.00	
In Poverty	93.5%	6.6%		1.14 (0.42, 3.11)	0.80
***Health Variables***					
**Lifetime Smoking**					
Less than 100 Cigarettes (Ref.)	94.6%	5.4%	0.0009	1.00	
100 or more Cigarettes	85.9%	14.1%		1.86 (0.76, 4.54)	0.18
**Nativity**					
US-born (Ref.)	92.7%	7.3%	0.38	1.00	
Foreign-born	90.0%	10.0%		1.48 (0.63, 3.48)	0.37
**Exercise**					
Less than Moderate Exercise	92.8%	7.2%	0.64	0.72 (0.37, 1.40)	0.34
Moderate Exercise (Ref.)	91.6%	8.5%		1.00	
**Body Mass Index (BMI)**					
BMI< 25 (Ref)	93.7%	6.3%	0.048	1.00	
BMI >=25, <30	88.3%	11.7%		1.77 (0.89, 3.55)	0.11

**†** Age was divided by 10 and can be interpreted as odds for diabetes by decade. All variables in the table were included in the logistic regression analysis.

**Table 3:** Diabetes management behaviors of nonobese Japanese-American and non-Hispanic White cohorts (n=7,739).

Variable Name	Japanese American † (n = 269)	Non-Hispanic White † (n = 7,470)	p-value
Eye Exam Regularly Done	**95.2**%	**87.1**%	**0.002**
Foot Exam Regularly Done	69.6%	73.4%	0.67
A1C Check Regularly Done	73.8%	69.3%	0.58
Confident to Control & Manage Diabetes†	95.9%	94.7%	0.71

† The sample size for “Confident to Control & Manage Diabetes” was different from optimal eye exam, optimal foot exam, and optimal A1C check. The total sample was 6063, with 217 Japanese Americans and for 5846 non-Hispanic Whites.

**Table 4:** Logistic regression of diabetes management variables for non-Hispanic White vs. Japanese-American adults with BMI < 30.

	Crude (Unadjusted) OR (95% CI)	Model 1 OR (95% CI)
(a) *Optimal Eye Care (n = 7,739)*		

White (Ref: Japanese)	0.34 (0.17, 0.68)*∗∗*	0.35 (0.17, 0.74)*∗∗*

(b) *Optimal Foot Care (n = 7,739)*		

White (Ref: Japanese)	1.21 (0.50, 2.91)	1.21 (0.50, 2.94)

(c) *Optimal Testing for A1C Levels (n = 7,739)*	0.80 (0.35, 1.82)	0.77 (0.34, 1.79)

White (Ref: Japanese)		

(d) *Confidence to Control and Manage Diabetes (n = 6,063)*	0.78 (0.13, 4.74)	1.04 (0.17, 6.38)

White (Ref: Japanese)		

*∗*p<0.05, *∗∗*p<0.01, and *∗∗∗*p<0.001.

Crude includes ethnicity only in the analysis.

Model 1 includes ethnicity, insulin dependence, age, sex, education, poverty, and overweight( (BMI 25 to 29.99) *vs* BMI<25).

## Data Availability

Data for the California Health Interview Survey (CHIS) is available publicly from the UCLA Center for Health Policy Research. CHIS is funded by a network of public agencies and private foundations, and Public Use Files (PUFs) have been made available starting in 2001.

## Additional Points

*Key Messages.* (1) Asian-Americans are the fastest growing ethnic/racial minority group in the United States, yet they remain underrepresented in most epidemiological studies. (2) Previous studies show that there are significant differences in health outcomes and disease prevalence between Japanese-Americans and their Asian and non-Hispanic White counterparts. (3) In this study, the prevalence of diabetes among nonobese Japanese-American adults was higher than their non-Hispanic White counterparts (8.0% versus 4.5%). (4) One quarter of nonobese Japanese Americans aged 80 and older have diabetes. (5) There is a need to develop appropriate intervention and prevention approaches with lifestyle modification specifically targeted towards nonobese Japanese-Americans.
